# Hypodermic Medication

**Published:** 1890-10

**Authors:** 


### Book Reviews.

---

HYPODERMIC MEDICATION. By Bourneville & Bricon. Revised and Edited by G. Archie Stockwell, M. D., F. R. S. "The Physician's Leisure Library." Geo. S. Davis, Publisher, Detroit, Mich., 1890. Price, in paper, 25 cents—in cloth, 50 cents.

This little book contains 125 pages of excellent matter. It is ably revised and edited. The alphabetical arrangement of both the text and index, indicates the editor's thorough conception of what goes to make a perfect book. This work is concise, and yet complete, and is well up to date. It treats of all medicines, old and new, that may be used hypodermically; mentions the diseases for which each is applicable—giving the quantity to be administered, and formula for the preparation of solution.

We congratulate the revisor and editor, upon the success of his labors, and heartily recommend the volume to the profession, as being one that meets all the requirements of this important branch of therapeutics.

It is very neatly bound in cloth and paper—by Geo. Davis & Co. E. V. J.

